# eIF4E as a Control Target for Viruses

**DOI:** 10.3390/v7020739

**Published:** 2015-02-16

**Authors:** Hilda Montero, Rebeca García-Román, Silvia I. Mora

**Affiliations:** 1 Public Health Institute, University of Veracruz, Veracruz 91190, Mexico; E-Mail: garciaromanrebeca@gmail.com; 2 Preparative Procedures and Access to Proteomic Services Unit, Institute of Biomedical Research, National Autonomous University of Mexico. Distrito Federal 04510, Mexico; E-Mail: sivonnemor@biomedicas.unam.mx

**Keywords:** eIF4E, 4EBP, virus, translation, latency, cancer

## Abstract

Translation is a complex process involving diverse cellular proteins, including the translation initiation factor eIF4E, which has been shown to be a protein that is a point for translational regulation. Viruses require components from the host cell to complete their replication cycles. Various studies show how eIF4E and its regulatory cellular proteins are manipulated during viral infections. Interestingly, viral action mechanisms in eIF4E are diverse and have an impact not only on viral protein synthesis, but also on other aspects that are important for the replication cycle, such as the proliferation of infected cells and stimulation of viral reactivation. This review shows how some viruses use eIF4E and its regulatory proteins for their own benefit in order to spread themselves.

## 1. Introduction

Until recently, the study of gene regulation used to focus on regulation at the transcriptional level. Nowadays, the translation process is known to be a target of the regulation of gene expression, which responds to a variety of conditions. Changes in one or more steps for controlling protein biosynthesis have been associated with alterations in growth regulation and cell cycle [1]. Consistent with this, it has recently been observed that translational control or translation machinery components are altered in pathologies such as cancer [2] and viral infections [3,4]. Even more recently, it has been found that there is a whole cellular regulation machinery through microRNAs (miRNAs) that inhibit or alter the translation of certain messenger RNAs (mRNAs) [5,6]. This is why, at last, great interest has been aroused in trying to understand the mechanism of cellular protein synthesis and its control.

The translation process is very complex and poorly understood. In recent decades, there has been progress in understanding both the mechanism and regulation of translation. It is known that transcriptional regulation may be important in gene expression. However, it has been determined that an increase in transcription does not necessarily lead to an increase in translation [7,8]. For this reason, research has been conducted in order to understand the mechanisms leading to the selection of transcripts that are translated more efficiently; yet so far, there has not been a precise answer to this question. The study of protein synthesis from viral mRNAs has made great progress in understanding the cellular translation and has also been used as a tool to understand how protein synthesis is regulated as well as the cellular proteins involved in the process.

Viruses need the machinery of their host cell for their genome replication and new particle assembly and release of new viral progeny. Whereas a number of viruses code for the enzymatic machinery required for replication and transcription of its genome, no virus that codes for components of the protein synthesis machinery has been identified [9]. This condition makes the virus totally dependent on the cellular machinery for their mRNA translation. Interestingly, viruses use strategies that favor the translation of their transcripts and their replication cycles by controlling components of the translational machinery.

## 2. Translation Mechanism

In the translation mechanism, diverse proteins known as translational factors are involved converting the information contained in the mRNA into a protein. This event is commonly divided into three phases: initiation, elongation, and termination [10,11,12]. The initiation phase has been described as the most regulated [10,11]; in eukaryotic cells, it can be carried out in two ways: (a) cap-dependent or conventional and (b) cap-independent or internal ribosome entry site (IRES) [10].

### 2.1. Cap-Dependent Translation Initiation

Most mRNAs of the cell are characterized by the m7GpppX structure (where X is any nucleotide) at the 5′ end, called cap. The 3′ end of mRNA contains a polyadenylated tract (poly-A), which is attached to the poly-A-binding protein (PABP). Both cap and poly-A have been observed to play key roles in translation efficiency [10,13,14].

**Figure 1:**
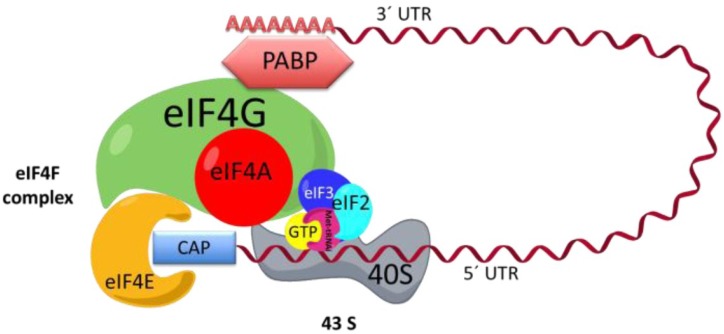
Initiation complex. The interaction between the eIF4F complex, 43S, and mRNA is shown. EIF4F is formed by eIF4A, eIF4G, and eIF4E. The complex 43S is formed by eIF3, the small ribosomal subunit, and eIF2, which in turn is formed by methionine-tRNA-initiator (Met-tRNAi) and GTP. The mRNA is recruited to the eIF4F complex across the interaction of the 3′ end and poly-A-binding protein (PABP) and the 5′ cap and eIF4E. UTR: untranslated region.

In the cap-dependent mechanism, to translate an mRNA, it is important that the mRNAs be recruited in a protein complex called eIF4F, which is composed of three proteins: the eIF4E protein, which binds to cap [15], and helicase eIF4A and eIF4G (Figure 1), which in turn bind to eIF4E, eIF4A, and PABP [16,17,18]. 43S is another complex involved in the initiation phase. 43S is formed by the small ribosomal subunit 40S, the factor eIF3, and ternary complex, the latter of which is formed by eIF2, GTP, and the methionine-tRNA-initiator (Met-tRNAi). It has been proposed that the eIF4F complex enlists the 43S complex via the interaction between eIF3 and eIF4G (Figure 1) [17,19].

The ribosomal subunit 40S carries the eIF2-GTP-Met-tRNAi complex to the start codon, where both ribosomal subunits 40S and 60S blend to form the complete ribosome 80S [10,11,12]. eIF2 is released along with GDP. The GDP bound to eIF2 is exchanged for GTP by the eIF2B factor to start a new initiation round [16,17,20,21].

### 2.2. Cap-Independent Translation Initiation

It is suggested that the cap-independent translation mechanism occurs when cap-dependent translation is limited [22]. This alternate initiation proposes that a complex secondary structure in the 5′ untranslated region (5′ UTR) called IRES is important for translation of mRNA. The IRES mechanism was initially described in the picornavirus family [23,24]. However, it is now known that the IRES is not unique to viral mRNAs, as it has been found that IRES-containing cellular mRNAs code principally for proteins involved in cell recovery from stress conditions [25,26] and during the cell cycle [27]. Interestingly, in this initiation form, an mRNA with IRES can be translated with or without the requirement of any canonical initiation factor [28] or can use cellular proteins known as IRES trans-acting factors (ITAFs) that function as cofactors to facilitate or encourage translation [29,30,31].

## 3. eIF4E as the Translational Control Target

A variety of cellular proteins are involved in the translation process. Although all of them are important during this process, one of the main regulation points involves eIF4F complex formation via eIF4E.

Three mechanisms have been found by which eIF4E can control eIF4F complex formation and interfere with cellular translation [32]. One of these mechanisms is via phosphorylation on serine 209 and is carried out by the mitogen activated kinases (Mnk1 and Mnk2). This kinase is recruited in the eIF4F complex to phosphorylate eIF4E via its interaction with eIF4G. It has been suggested that this strategy ensures that eIF4E is phosphorylated only when it is a part of the eIF4F complex [3,10,18,19,33]. In mammals, translational stimulation by growth factors, nutrients, or serum is correlated with increased eIF4E phosphorylation, whereas eIF4E dephosphorylation is correlated with the inhibition of cap-dependent translation under heat-shock conditions, lack of nutrients, or some viral infections [18,34].

A second regulation mechanism on eIF4E is via 4EBPs (eIF4E-binding proteins). These proteins are considered translational repressors as they bind and hijack the eIF4E factor, preventing it from binding to eIF4G. Three types of 4EBP proteins have been described: 4EBP-1, 4EBP-2, and 4EBP-3; but the importance of the diversity of these proteins is unknown. Of the three types of 4EBPs, 4EBP-1 is the best characterized and is known to be a phosphoprotein and its binding to eIF4E depends on its phosphorylation status [35,36]. When the 4EBP-1 protein is hyperphosphorylated by mammalian target of rapamycin (mTOR) kinase, it cannot bind to eIF4E and the eIF4F complex is functional. Conversely, when these proteins are hypophosphorylated, they bind to eIF4E, avoiding its interaction with eIF4G. Thus, the eIF4F complex cannot be formed [3,18,37].

Finally, a third regulation mechanism is related to the abundance of eIF4E [32], which is the least abundant initiation factor, so this feature makes eIF4E a limiting factor in eIF4F complex formation. eIF4E is a transcriptional target of c-Myc [38]; interestingly, overexpression of eIF4E has been observed to cause malignant transformation of human epithelial cells and fibroblasts and promote tumor formation in transgenic mice [39,40]. Consistent with the oncogenic potential of eIF4E, several studies have shown that human tumors express high levels of this protein, and it has been particularly interesting to study the regulation of this factor in the control of these diseases [41,42,43]. Use of rapamycin as a therapeutic agent in the development of cancer has been studied since this drug specifically inhibits mTOR kinase by causing 4EBP hypophosphorylation, resulting in eIF4E hijacking [1,2,37]. Similarly, Chang and colleagues [44] developed a recombinant adenovirus-associated virus that can be administered to mice via aerosol. This virus expresses 4EBP-1 and interferes with the oncogenic function of eIF4E, inhibiting the proliferation of cancer cells with promising results [44].

## 4. eIF4E Regulation During Viral Infections

Viruses of different families are known to be capable of regulating cap-dependent translation by altering eIF4F complex translation initiation. In these cases, the viral mRNAs are translated efficiently by initiation mechanisms different from those observed for cellular mRNAs [45]. Some viruses employ translation initiation mechanisms in which they do not use or reduce the initiation factors of the eIF4F complex; in most cases, these viruses inactivate one or more components of this protein complex, one of the main targets of which is eIF4E [45,46]. Viral regulation exerted on this initiation factor may confer advantages to the viruses, such as promoting their protein synthesis, reactivating latency, or stimulating cell proliferation, which are necessary conditions to complete the replication cycle.

### 4.1. eIF4E Regulation via Phosphorylation at Serine 209

**Figure 2:**
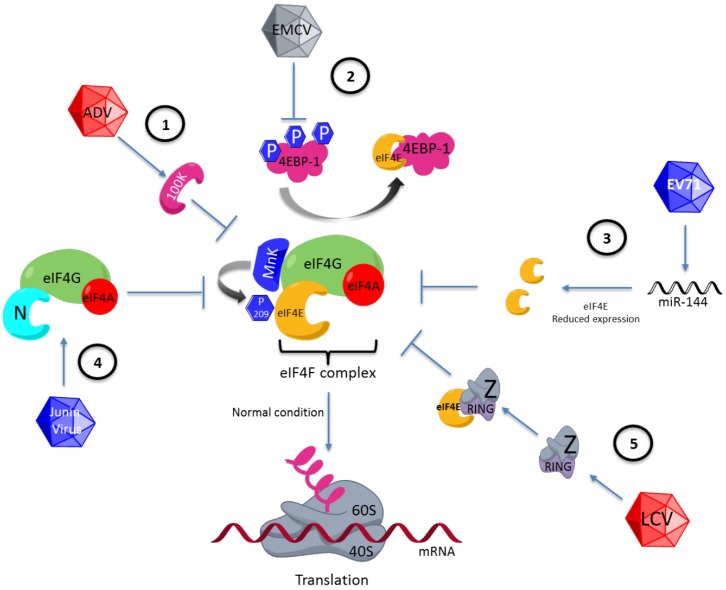
Differential regulation of eIF4F by various viruses. (1) eIF4E regulation via phosphorylation at serine 209. eIF4E is regulated by dephosphorylation by blocking of MnK1 by 100K adenovirus viral protein. (2) Regulation on 4EBP1. 4EBP1 is dephospohorylated during the encephalomyocarditis virus (EMCV) infection and eIF4E is hijacking the by 4EBP1 protein. (3) Alteration in eIF4E expression. Enterovirus 71 (EV71) reduces eIF4E expression by inducing the synthesis of microRNA 141 (miRNA-141). (4) Replacement of the function of eIF4E by viral proteins. Junin virus codifies the N viral protein, which substitutes for eIF4E of the eIF4F complex. (5) eIF4E regulation via its binding to viral proteins. The Z lymphocytic choriomeningitis virus protein is joining to eIF4E across the RING (really interesting new gene) motif, blocking the eIF4E function. LCV: lymphocytic choriomeningitis virus; ADV: adenovirus.

One example of viral translation regulation via eIF4E is observed during adenovirus infection. This virus inhibits the cap-dependent translation used by cellular mRNAs and efficiently promotes viral mRNA translation in the late phase of infection by using a mechanism involving eIF4E dephosphorylation. This event has been studied extensively, and the genome of this virus has been found to code for a protein called 100 K, which binds to eIF4G, preventing binding of this factor to eIF4E kinase, MnK1. Thus, the eIF4E factor is not phosphorylated, inhibiting cellular protein synthesis (Figure 2). Moreover, mRNAs of adenovirus are efficiently translated since they contain a leader sequence located at the 5′ UTR, a characteristic of all late-phase viral mRNAs. This sequence has been proposed to be capable of enlisting the complex 43S by base pairing between the viral mRNA and the rRNA of the 40S ribosomal subunit [47,48]. This form of translation is an example in which a mechanism similar to that observed in prokaryotes is used; in this microorganism, the Shine-Dalgarno sequence (UCCUCCA) is important for translation [49].

It has been found that, in cells infected by the influenza virus, there is a decrease in cellular transcription; also, there is a selective degradation of cellular mRNAs in addition to eIF4E dephosphorylation [50]. These events have been suggested to lead to the inhibition of cellular protein synthesis. For translation of viral mRNAs, which are very similar in structure to cellular mRNAs, involvement of both eIF4A and eIF4G bound to the subunit PB2 of the viral polymerase is necessary [51].

### 4.2. Regulation on 4EBP

The viruses can regulate to eIF4E through 4EBPs. A well-characterized example has been observed in cells infected by encephalomyocarditis virus (EMCV), in which the 4EBP-1 protein is dephosphorylated, enabling its interaction with the eIF4E factor, and consequently the eIF4F complex is not formed (Figure 2) [52]. The mRNAs of EMCV have IRES structures that enable efficient translation independently of eIF4F complex formation [53,54]. Another example is observed in the cells infected by the vesicular stomatitis virus (VSV), which induces eIF4E and 4EBP-1 dephosphorylation. These two events are correlated with the inhibition of cellular protein synthesis observed during infection with VSV [55].

Interestingly, there are cases in which the viruses positively regulate eIF4F complex formation via 4EBP-1. It was found that, during infection with herpes simplex virus (HSV), the viral gene product ICP0 promotes degradation of 4EBP-1 via proteasome, avoiding interaction with eIF4E. Moreover, the eIF4E factor was found to be phosphorylated within infected cells. This phosphorylation favors viral replication since inactivation of MnK1 kinase causes decreased translation and viral replication, suggesting that eIF4E phosphorylation by MnK1 is important in these events. Although during infection with HSV the eIF4F complex formation is favored, preferential synthesis of viral proteins compared with cellular protein synthesis is observed. The mechanism by which mRNAs of this virus are preferentially translated is not fully understood. However, the pool of cellular mRNAs is known to be depleted in cells infected with HSV, creating the possibility that the efficient synthesis of viral protein is a result of the relative abundance of viral mRNA compared to cellular mRNAs [56]. Conversely, during latency of this virus in neurons, inhibition of eIF4F formation, via disruption of the mTOR-4EBP-eIF4E pathway, has been found to be directly involved in the reactivation of the latency of HSV as the initial signal of the viral gene expression program [57]. This is the first case in which the control of a translational pathway can be a switch between the latency and the reactivation of a virus and is an example of the same pathway exerting an influence differentially during different stages of the viral replication cycle.

Another virus that stimulates eIF4F formation is the hepatitis C virus (HCV). This virus codes for a non-structural 5A protein with a kinase function whose phosphorylation target is 4EBP1, stimulating the cap-dependent translation [58]. This context could stimulate oncogenic activity of eIF4E, promoting cell proliferation and resulting in hepatocellular carcinoma. This idea is based on the fact that the virus does not stimulate cap-dependent translation to favor its protein synthesis, as the translation of viral mRNAs is carried out via IRES and the eIF4F complex is not required [59]. Interestingly, the base pairing between viral IRES of HCV RNA and rRNA 18S is important for enlisting the ribosomal subunit 40S [60]; a similar strategy is used by the mRNAs of adenovirus.

### 4.3. Alteration in eIF4E Expression

Enterovirus 71 (EV71) reduces eIF4E expression by inducing the synthesis of miRNA (miR-141), favoring cap-independent translation (Figure 2) [61]. If the activity of miR-141 is avoided and the eIF4E levels are maintained, the amount of virus generated decreases [62], suggesting that the depletion of eIF4E is important for the viral protein synthesis required for the formation of new virus or that eIF4E might interfere with the viral replication cycle. Therefore, EV71 uses a mechanism to prevent eIF4E expression. Hepatitis B virus (HBV) is an example in which eIF4E, like EV71, is involved in the viral replication cycle but in a process different from that of translational control. eIF4E has been shown to form a complex with both the viral polymerase and the 5′ stem-loop structure of the HBV genome [63]; interestingly, eIF4E is introduced in the viral particle. eIF4E has been suggested to play a role in the viral morphogenesis or retrotranscription [64].

The Epstein-Barr virus has been associated with the development of cancer [65,66] and, unlike EV71, induces eIF4E overexpression [67]. The replication mechanism of this virus is very interesting. One of the viral proteins, latent membrane protein 1 (LMP1), has been proposed as a viral oncogene. LMP1 achieves increased eIF4E expression via c-Myc, and the consequent cell proliferation results in development of nasopharyngeal carcinoma [67]. This mechanism is consistent with the observed role of eIF4E in the development of cancer, whose mechanism of action is possibly used by a virus to favor cell proliferation and the spread of the virus.

### 4.4. Replacement of the Function of eIF4E by Viral Proteins

Some viruses promote their protein synthesis via the replacement of eIF4E. In the Junin virus, the viral N protein replaces eIF4E in the eIF4F complex (Figure 2) [68]. Furthermore, infection with HIV-1 promotes the binding of DEAD-box helicase to eIF4G and PABP, so that eIF4E is displaced and the genome of this virus can be efficiently translated [69]. There is an example in which not only the function of eIF4E is replaced but the function of the eIF4F complex is assumed by the viral protein of the nucleocapsid N of the hantavirus [70], showing the multifunctional role assumed by some viral proteins in order to have enough viral protein to form new viruses.

### 4.5. eIF4E Regulation via Its Binding to Viral Proteins

There is an alternate way for regulating eIF4E during viral infections by the direct interaction of this factor with different proteins coded by viruses. The lymphocytic choriomeningitis virus codes for the Z protein, which has the characteristic of binding to eIF4E and blocking its function by reducing its binding to cap (Figure 2). The bond between eIF4E and Z protein is through a RING (really interesting new gene) motif located in a region different from those of eIF4G and 4EBP [71]. Otherwise, there are viral proteins that bind to eIF4E in order to stimulate its function as a translational factor, as in the case of sapovirus, which codes for a viral protein known as VPg. This protein binds to the viral genome and eIF4E, favoring viral protein synthesis [72].

## 5. Conclusions

Viruses use the cellular machinery to generate progeny because they do not code in their genomes for all of the components required for replication. eIF4E is a key protein in translational regulation and is involved in diverse cellular processes such as proliferation. Recent studies show that eIF4E is a common target for some viruses, which regulate it to benefit their replication cycles, where eIF4E not only plays important roles in the translation of viral mRNAs but also is involved in the proliferation of infected cells (associated with the development of cancer) and the reactivation of viral latency. There is still much to be learned about each replication cycle, but it is clear from the current knowledge of eIF4E in viral mRNA translation that the mechanisms and viral proteins involved in their regulation are very important. These areas are now being studied to control viral infections and other diseases such as cancer [44,73].
